# Unilateral High Brachial Artery Bifurcation Encountered During Cadaveric Dissection: An Anatomical Case Report

**DOI:** 10.7759/cureus.111140

**Published:** 2026-06-19

**Authors:** Dheenadhayalan MJ, Roshini N, Kalpana Ramachandran

**Affiliations:** 1 Anatomy, Sri Ramachandra Institute of Higher Education and Research, Chennai, IND

**Keywords:** anatomical variant, arterial variation, brachial artery, cadaveric dissection, upper limb

## Abstract

Any difference in the pattern of branching of the brachial artery has clinical significance, that is, they possess surgical significance such as the formation of a hematoma during ligation and vascular grafting. Another significant rare anatomical variant that is important for clinicians to know is the high branching of the brachial artery.

An unusual or high bifurcation of the brachial artery was seen in the superior one-third of the arm of the right upper extremity of an adult female cadaver during regular cadaveric dissection. Using the ruler method, it was measured that the brachial artery bifurcated into the radial and ulnar arteries at 4.5 cm distal to the point of insertion of the teres major muscle (far above another common site of bifurcation in the cubital fossa). The median nerve travelled between both terminal branches. The radial artery followed the downward course of the arm at a lateral position, whereas the ulnar artery followed the same path at a medial position. The profunda brachii artery branched off the brachial artery, although it did so prior to the brachial artery dividing into two. On examination of the left upper limb, it was evident that the brachial artery was normal and divided into two branches at the cubital fossa.

This case underlines the significance of the identification of anatomical differences in the arteries of the upper extremity. High bifurcation of the brachial artery has significant clinical implications; knowledge of this brachial artery variation is important for surgeons performing arterial cannulation and vascular access procedures of the upper limb, as well as for interventional radiologists, anesthesiologists placing arterial lines, and clinicians interpreting vascular imaging studies. Knowledge of such variations is crucial to avoid iatrogenic injuries and ensure successful clinical outcomes. Preoperative imaging and anatomical knowledge are crucial in the process of discovering these variants.

## Introduction

The brachial artery is a major blood supply to the upper extremity, which is an extension of the axillary artery at the inferior edge of the teres major muscle [1]. Traditionally, the brachial artery travels along the arm in the medial bicipital groove between the biceps brachii and triceps brachii on the medial aspect of the arm. It contains the brachial artery and median nerve, with the median nerve crossing the brachial artery from lateral to medial in the mid-arm, with the median nerve and basilic vein, and ends by bifurcating into radial and ulnar arteries at the cubital fossa level about 1 cm beneath the humerus intercondylar line [1].

The branching pattern of the brachial artery has been widely shown in both anatomical and clinical literature, with prevalence rates of 1%-20% depending on the particular variation and population sample [2-4]. These arise due to defects in the vasculature of the upper limbs during embryonic development, specifically retention or loss of portions of the basic axial artery and capillary plexus [5,6].

One such clinically significant variation is the high bifurcation of the brachial artery, where the division takes place above the cubital fossa. This anomaly has significant implications for surgical interventions to the arm and forearm, arterial catheterization and cannulation, measuring blood pressure, diagnosing and managing compartment syndrome, vascular imaging studies interpretation, and upper-limb trauma management [7-10].

Such variations have an embryological basis in the complicated development of the upper extremity arterial system. During the fifth week of embryonic development, the axial artery emerges as the primary arterial channel supplying the developing upper limb bud [5]. This primitive vessel evolves into a capillary plexus to which the ultimate arterial pattern is created as a result of selective preservation and regression [6]. The process may be subject to variations that can lead to early bifurcation, retention of superficial vessels that regress, or aberrant regression patterns [5,6].

Some systems for classification have been proposed for brachial artery variations, such as the historical Adachi classification [2], the comprehensive Lippert and Pabst classification [11], and the modern embryologically based classification by Rodríguez-Niedenführ and colleagues [4,6]. These systems assist in classifying variations in terms of origin, course, and relation to the nearby structures.

This case report involves a high division of the brachial artery at 4.5 cm at the bottom of the teres major insertion that was found during routine anatomical dissection. The arterial pattern of the upper limb develops from the primitive axial artery during the fifth week of embryogenesis. Variations such as high bifurcation of the brachial artery may result from the persistence, regression, or abnormal remodeling of embryonic vascular channels during development.

## Case presentation

Cadaveric specimen

An anatomical variation was detected during normal anatomical dissection of a cadaver of an adult female in the course of undergraduate medical education in the dissection of the right upper extremity. The cadaveric specimen was preserved using a standard formalin-based embalming solution according to the institutional protocol. The donor had given informed consent for the use of their corpse in the anatomy learning and investigation.

Dissection findings

**Figure 1:**
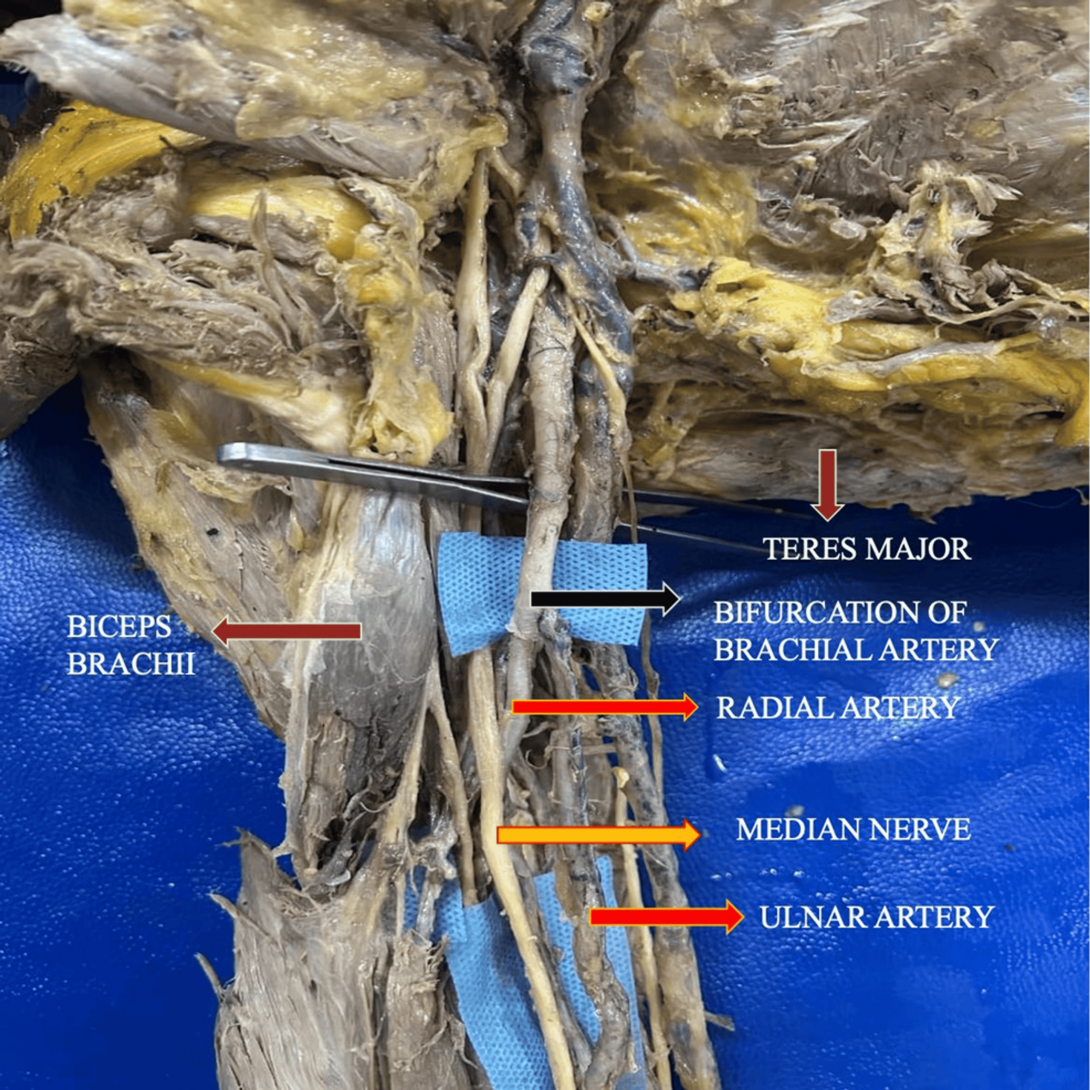
Anterior view of the right arm demonstrating high bifurcation of the brachial artery in the upper one-third. The probe (black) marks the bifurcation point located 4.5 cm distal to the teres major (TM) insertion. The radial artery (RA) courses laterally while the ulnar artery (UA) courses medially. The median nerve (MN) is visible coursing between the two terminal branches. The biceps brachii muscle has been reflected to expose the neurovascular bundle.

The anterior part of the right arm was dissected. The skin and superficial fascia of the arm were carefully reflected, followed by the removal of the deep fascia. The biceps brachii muscle was exposed, and the neurovascular structures within the medial bicipital groove were identified. The brachial artery and its branches were meticulously dissected and traced from the lower border of the teres major muscle to the cubital fossa. During this procedure, a high bifurcation of the brachial artery was observed and documented (Figure 1).

Location and morphology

The brachial artery is separated in the upper third of the arm, 4.5 cm distal to the origin of the brachial artery at the inferior border of the teres major muscle, corresponding to the level of the teres major insertion. This located the bifurcation point well proximal to the normal bifurcation site at the cubital fossa, which normally is about 1 cm below the intercondylar line of the humerus. The split was made at about the point of the deltoid muscle insertion on the humerus, which is in one-third of the arm.

Branch of arteries and course

**Figure 2:**
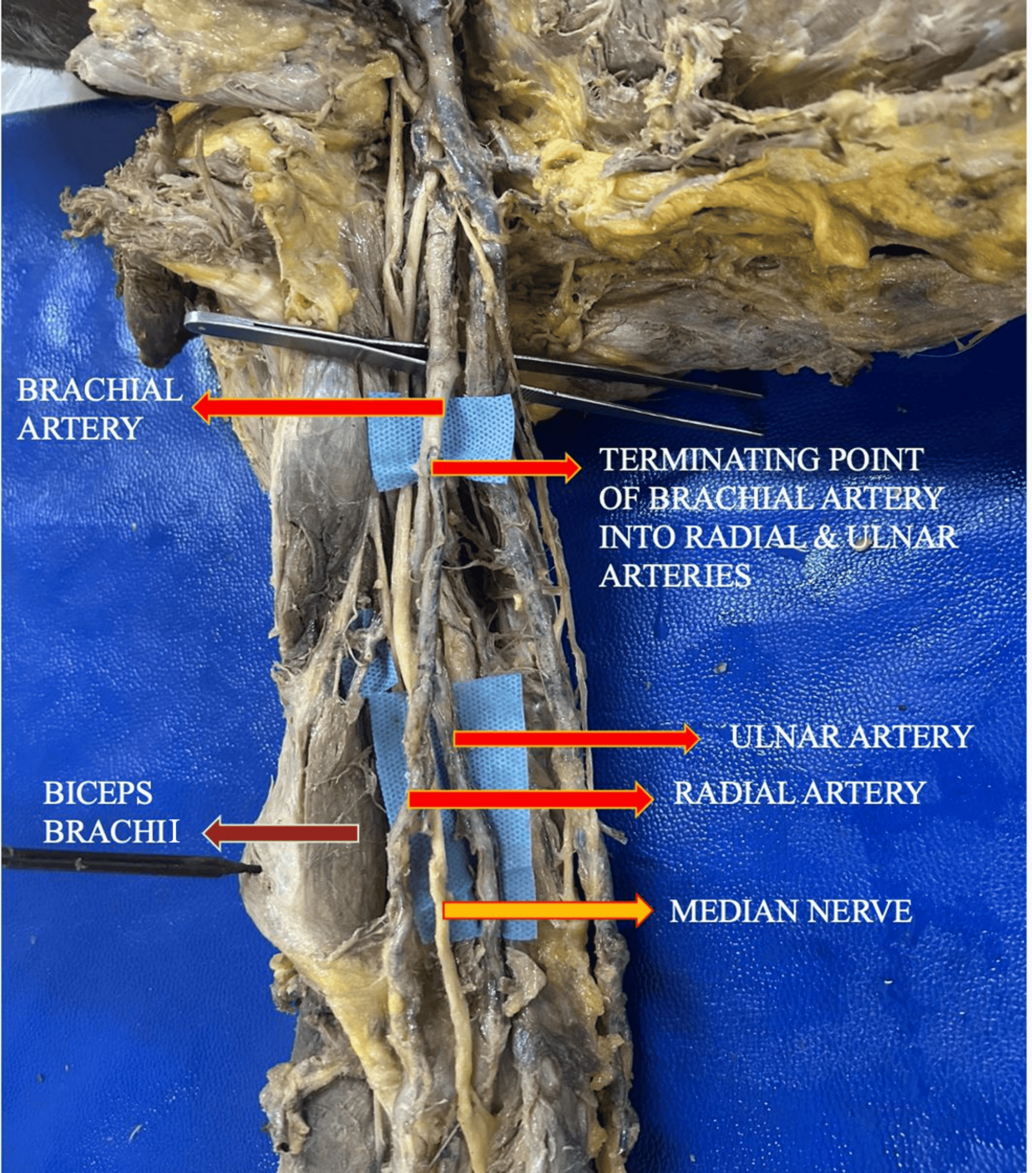
Close-up view of the bifurcation point showing the anatomical relationship between the arterial branches and the median nerve (MN). The bifurcation is clearly visible with the probe marking the division site. Multiple blue markers delineate the course of both terminal vessels. The intimate relationship of the median nerve to the bifurcating vessels is demonstrated, with the nerve positioned between the radial and ulnar arteries throughout their course in the arm.

**Figure 3:**
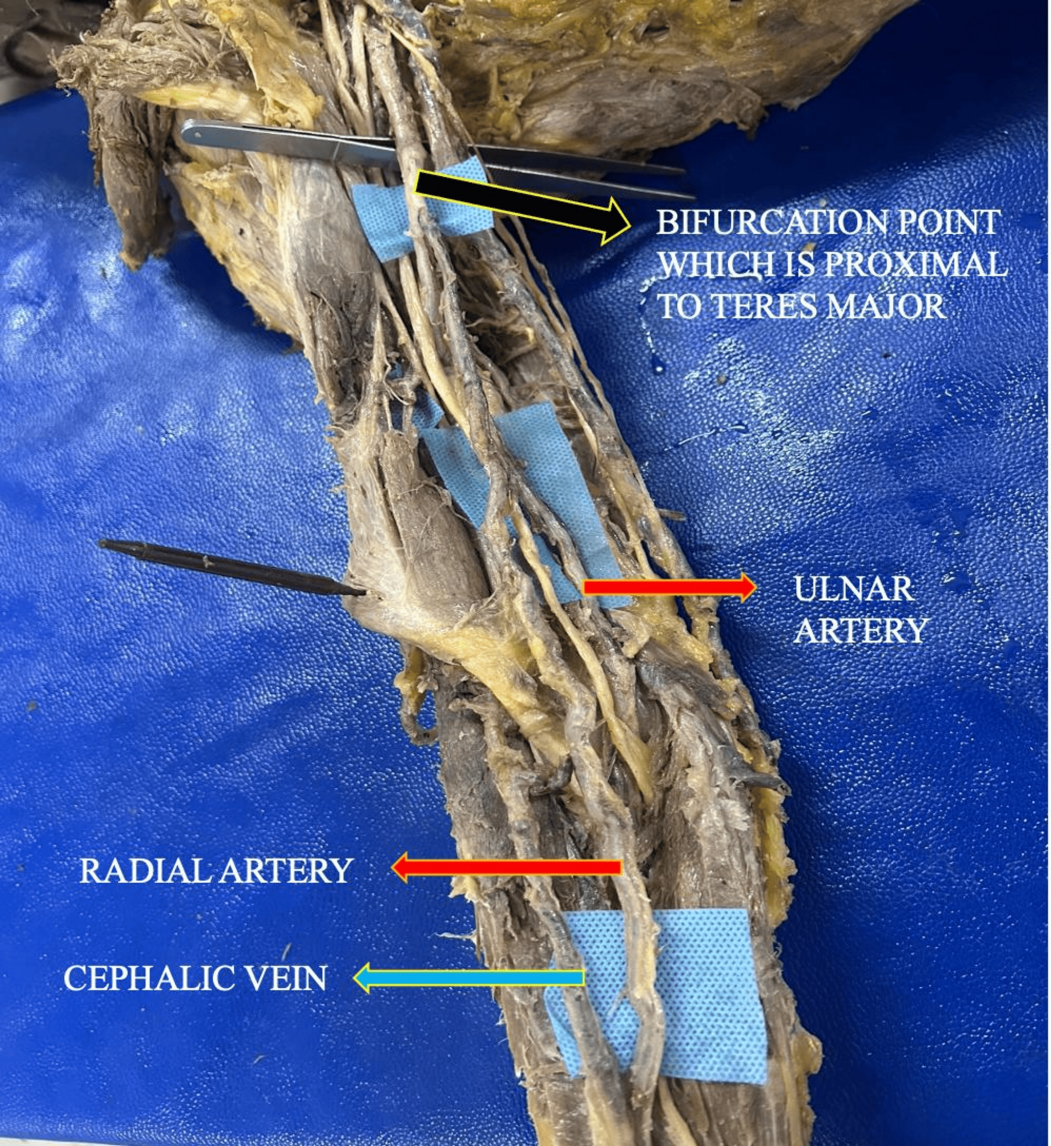
Detailed view showing the distal course of the radial and ulnar arteries as they descend through the arm toward the elbow. The probe marks the bifurcation point proximally. The separated arterial branches maintain their respective lateral (radial) and medial (ulnar) positions. The distance of 4.5 cm from the teres major insertion to the bifurcation point places this variation in the upper one-third of the arm.

The artery divided into two terminal branches, which would be known as the radial and ulnar arteries depending on their future anatomical path, diameter, and association with other structures (Figures 1-3). In addition to the high bifurcation of the brachial artery, the radial artery exhibited a mildly tortuous course in the arm before continuing distally into the forearm, following a lateral course down the arm, keeping a very superficial course as it continued to the forearm. It then went to the median nerve and proceeded along the lateral aspect of the anterior compartment. The ulnar artery followed a medial course along the arm, crossing the median nerve and heading down toward the medial epicondyle. It remained near the median nerve as it ran in the arm.

Association with the median nerve

At the bifurcation, the median nerve was found to run between two end branches (Figure 2). The nerve was in its normal location in the anterior arm compartment and was between the radial and ulnar arteries instead of serving the brachial trunk. This correlation extended all along the arm as far as the bifurcation point to the cubital fossa.

Additional branches

The profunda brachii artery (deep artery of the arm) was developed out of the brachial artery before the point of bifurcation, retaining the usual root and path. The higher and lower ulnar collateral arteries seemed to originate from the ulnar artery following bifurcation as opposed to the main brachial trunk, which is also another variation to the normal arrangement.

Contralateral comparison

Left upper limb dissection showed the normal structure of the brachial artery including regular bifurcation of the artery at the position of the cubital fossa, which is about 1 cm below the intercondylar line of the humerus. This proved that the variation was unilateral and was confined to the right upper limb.

Measurements and classification

During dissection, the following measurements were taken: the distance from the lower border of the teres major (insertion point) to the site of bifurcation was 4.5 cm. The variation was present on the right aspect only. Based on the morphological features, the bifurcation was a simple division into the radial and ulnar arteries, corresponding to a Type I pattern according to conventional classification systems [4]. No accessory vessels or other arterial anomalies were found.

Photographic documentation

Three quality photos were acquired, capturing the anatomical variation of the various angles (Figures 1-3). The pictures illustratively depict the point of bifurcation where a probe is placed, the direction of the two end branches, and their connection with the median nerve. The images depict the disembodied anterior part of the right arm with the neurovasculature exposed and marked with blue marks to identify it easily.

## Discussion

Embryological basis

During the fifth week of intraembryonic development, the peripheral division of the seventh intersegmental artery becomes the arterial system of the upper extremity [5,12]. The limb bud is first fed by a primitive axial artery that runs along the growing limb. This primitive vessel is then replaced by a capillary plexus out of which an ultimate arrangement of arteries develops through a process of selective persistence and regression [5,6].

The normal developmental pattern has the axillary artery develop out of the seventh intersegmental artery, the brachial artery out of the axial artery, and the radial and ulnar arteries out of the terminal bifurcation of the axial artery at the region of the cubital fossa [5,6,13,14]. Deterministic and stochastic variations can lead to early bifurcation, with premature division of the axial artery leading to high brachial bifurcation, retention of superficial capillary channels that normally regress, or abnormal regression patterns such as loss of segments that would normally survive [5,6,15].

The bifurcation seen at this location of 4.5 cm from the teres major insertion was probably caused by the early separation of the rudimentary axial artery and the development of in-between segments by normal regression. This is in line with embryological accounts of the development of upper extremity arteries as established by Rodríguez-Niedenführ and others in their detailed studies of the development of the arteries [5,6].

Classification and prevalence

A number of classification systems have been suggested in relation to variations of brachial arteries. One of the first comprehensive classifications by cadaveric studies of Japanese populations was the Adachi classification [2]. The Lippert and Pabst classification [11] classified variations by origin and course, which offered a systematic way of describing arterial anomalies. The Rodríguez-Niedenführ et al. classification [4,6] is a more recent classification that takes into account the embryological patterns and gives a clue about the developmental mechanisms of these variations.

The literature has reported high bifurcation of the brachial artery with different prevalence. Cadaveric studies indicate a prevalence of 1%-20% with definition and population of study [3,4,9,10]. Angiographic research reveals 2%-10% of cases exhibit early bifurcation [16,17]. Geographic and ethnic differences have been observed, with certain groups exhibiting greater prevalence [9,18,19].

The variation can also be further categorized as Type I (high bifurcation of the radial and ulnar arteries, as in our case), Type II (high bifurcation with accessory vessels), or Type III (trifurcation patterns) [4,16]. Our case can be considered a classic Type I pattern in which the bifurcation is at 4.5 cm distal to the insertion of the teres majora.

Such variations have been recorded in recent researches. Yang et al. [20] presented a case of high bifurcation with a focus on unilateral presentation. Jangir et al. [21] reported a unique high bifurcation with clinical consequences. Glin et al. [22] carried out a systematic review of literature to assess morphological changes and their clinical implications in various studies.

Clinical implications

Surgical Considerations

In case of upper extremity trauma, it is important to know of any potential variation to prevent unintentional ligation or injury in the course of achieving hemostasis or repair of vessels [23,24]. Orthopedic surgeries of the humerus, like fracture repair or tumor removal, may encounter an anomalous vessel in a surprising position [25]. Plastic and reconstructive surgeons who create flaps to be used in microsurgical reconstruction need to have proper vascular mapping to ascertain the viability of flaps [26]. Surgeons involved in embolectomy, thrombectomy, or arterial repair surgeries may be involved in anatomical surprises [17,23].

Anesthetic implications

Hemodynamic monitoring with arterial line placement can lead to the cannulation of an end branch (instead of the main trunk), and this can contribute to errors in blood pressure measurements [24,27]. The use of regional anesthesia procedures and methods, especially nerve blocks with ultrasound in the upper arm, involves significant attention to the identification of the vascular structures to prevent intravascular injection [27]. The presence of two arterial channels in the proximal arm can influence the placement of a tourniquet and the need to ensure sufficient compression of the arteries [24].

Diagnostic considerations

In case the cuff squeezes only one of the terminal branches, the blood pressure measurements can be erroneous and thus give false results [24,27]. Doppler ultrasound research must be conscious of potential variability so that it can be read correctly [17]. Variations need to be detected using angiography and other preoperative imaging modalities to determine the surgical or interventional procedure [16,17].

Clinical examination

Palpation of the pulse can be challenging or even non-existent at anticipated points when the artery has bifurcated before the point of examination [10,24]. The test to evaluate collateral circulation to the hand could have changed the interpretation in the variant anatomy [10]. Multiple arterial channels, which can cause different compartment pressures, may complicate the assessment of compartment syndrome [24].

Dialysis access creation

The development of arteriovenous fistulas to access hemodialysis in the upper limb should be done cautiously by taking into consideration the arterial anatomy. High bifurcation might influence the selection of the access site and can be a complication to the maturation of the fistula [27].

Comparison against published literature

Earlier cadaveric research has found high brachial bifurcation with varying frequencies and different levels of prevalence in different populations. One of the most extensive studies was carried out by McCormack et al. [3], who examined 750 extremities and recorded the different arterial patterns. Korean cadavers gave prevalence data [9], which had ethnic differences. The variations in the arteries of Turkish populations had been recorded by Yalcin et al. [19]. D'Costa et al. [18] and Kawashima et al. [26] described superficial arterial patterns in detail.

There are multiple case reports that report the clinical outcomes of unidentified high bifurcation. A case was reported by Cherukupalli et al. [23] that presented with acute arterial insufficiency. Bilodi and Sanikop [24] reported the clinically significant variations in the termination of brachial arteries. The importance of creating arteriovenous fistulas was discussed by Al Talalwah [27]. Ghouri et al. [17] offered general imaging advice on the assessment of upper extremity vasculature.

The Rodríguez-Baeza et al. [16], Natsis et al. [10], and Maslarski [28] classification and morphological studies have helped us to understand the range of variations. This information was summarized in recent extensive reviews and encyclopedic publications [29] to give practical guidelines to clinicians.

We had a bifurcation at 4.5 cm off the teres major insertion, which is within the range reported in the literature on high bifurcations, but reflects the extreme end of this range. The most common pattern [4,20,21] is consistent with the unilateral nature and Type I classification.

Clinical case reports and complications

A number of case reports have reported clinical effects of unidentified high bifurcation, such as unintended arterial injury during surgery on the arm [23,24], unsuccessful or problematic placement of arterial lines to monitor hemodynamic parameters [27], complications in creating dialysis access necessitating revision surgery [27], and misinterpretation of angiographic studies resulting in procedural complications [17].

These reports underscore the significance of preoperative imaging, especially ultrasound or angiography, in planning procedures in the upper limb. New imaging technologies, especially CT angiography and MR angiography, are capable of defining the structure of the arteries and identifying differences prior to treatment [17].

Limitations

This case report has a number of limitations. Being an isolated cadaveric observation, it has no clinical correlation with clinical implications or patient outcomes. We could not evaluate hemodynamic significance or compensatory mechanisms that can be present in living patients. There were no pre-mortem imaging studies (angiography, CT, or MRI) that could be compared to dissection results. The age at which death occurred and a comprehensive medical history of the cadaver were constrained or unknown, and it was impossible to correlate them with possible pathological conditions. There might have been an influence of the embalming process on the nature of the tissues, but there is no change in the significant anatomical relationships.

These drawbacks notwithstanding, cadaveric studies are still useful to record anatomical variations and to offer a basis for clinical knowledge. The intricate analysis it allows in cadaveric research offers data that cannot be easily discerned in clinical images.

## Conclusions

The present case describes a high bifurcation of the brachial artery occurring 4.5 cm inferior to the insertion of the teres major in the superior one-third of the right arm, with the median nerve passing between the radial and ulnar arteries. Although uncommon, such vascular variations have important clinical implications for surgeons, interventional radiologists, anesthesiologists, and other clinicians. Awareness of these variations is essential to prevent diagnostic errors and iatrogenic complications during surgical and vascular procedures. Preoperative imaging techniques such as USG, CT angiography, or MR angiography can aid in identifying these anomalies and facilitate safer procedural planning.

This case highlights that anatomical variations are not unusual and should be anticipated in clinical practice. A thorough understanding of vascular anatomy, its embryological basis, and meticulous surgical technique is crucial for safe and effective patient care. Continued reporting of such variations and comprehensive anatomical training will enhance knowledge of human anatomical diversity and contribute to better clinical outcomes.
